# Systemic β-Adrenergic Receptor Activation Augments the *ex vivo* Expansion and Anti-Tumor Activity of Vγ9Vδ2 T-Cells

**DOI:** 10.3389/fimmu.2019.03082

**Published:** 2020-01-24

**Authors:** Forrest L. Baker, Austin B. Bigley, Nadia H. Agha, Charles R. Pedlar, Daniel P. O'Connor, Richard A. Bond, Catherine M. Bollard, Emmanuel Katsanis, Richard J. Simpson

**Affiliations:** ^1^Laboratory of Integrated Physiology, Department of Health and Human Performance, University of Houston, Houston, TX, United States; ^2^Department of Nutritional Sciences, University of Arizona, Tucson, AZ, United States; ^3^Department of Pediatrics, University of Arizona, Tucson, AZ, United States; ^4^School of Sport, Health and Applied Science, St. Mary's University, London, United Kingdom; ^5^Department of Pharmacological and Pharmaceutical Sciences, University of Houston, Houston, TX, United States; ^6^Center for Cancer and Immunology Research, Children's National Health System and the George Washington University, Washington, DC, United States; ^7^Department of Immunobiology, University of Arizona, Tucson, AZ, United States

**Keywords:** exercise immunology, beta-blockers, gamma-delta t-cells, exercise, adoptive transfer immunotherapy

## Abstract

TCR-gamma delta (γδ) T-cells are considered important players in the graft-vs.-tumor effect following allogeneic hematopoietic cell transplantation (alloHCT) and have emerged as candidates for adoptive transfer immunotherapy in the treatment of both solid and hematological tumors. Systemic β-adrenergic receptor (β-AR) activation has been shown to mobilize TCR-γδ T-cells to the blood, potentially serving as an adjuvant for alloHCT and TCR-γδ T-cell therapy. We investigated if systemic β-AR activation, using acute dynamic exercise as an experimental model, can increase the mobilization, *ex vivo* expansion, and anti-tumor activity of TCR-γδ T-cells isolated from the blood of healthy humans. We also sought to investigate the β-AR subtypes involved, by administering a preferential β_1_-AR antagonist (bisoprolol) and a non-preferential β_1_ + β_2_-AR antagonist (nadolol) prior to exercise as part of a randomized placebo controlled cross-over experiment. We found that exercise mobilized TCR-γδ cells to blood and augmented their *ex vivo* expansion by ~182% compared to resting blood when stimulated with IL-2 and ZOL for 14-days. Exercise also increased the proportion of CD56+, NKG2D+/CD62L–, CD158a/b/e+ and NKG2A− cells among the expanded TCR-γδ cells, and increased their cytotoxic activity against several tumor target cells (K562, U266, 221.AEH) *in vitro* by 40–60%. Blocking NKG2D on TCR-γδ cells *in vitro* eliminated the augmented cytotoxic effects of exercise against U266 target cells. Furthermore, administering a β_1_ + β_2_-AR (nadolol), but not a β_1_-AR (bisoprolol) antagonist prior to exercise abrogated the exercise-induced enhancement in TCR-γδ T-cell mobilization and *ex vivo* expansion. Furthermore, nadolol completely abrogated while bisoprolol partially inhibited the exercise-induced increase in the cytotoxic activity of the expanded TCR-γδ T-cells. We conclude that acute systemic β-AR activation in healthy donors markedly augments the mobilization, *ex vivo* expansion, and anti-tumor activity of TCR-γδ T-cells and that some of these effects are due to β_2_-AR signaling and phenotypic shifts that promote a dominant activating signal via NKG2D. These findings highlight β-ARs as potential targets to favorably alter the composition of allogeneic peripheral blood stem cell grafts and improve the potency of TCR-γδ T-cell immune cell therapeutics.

## Introduction

TCR-γδ T-cells comprise 1–5% of the total peripheral blood T-cell pool and predominantly recognize non-classical MHC class I molecules and unconventional antigens such as phosphorylated microbial metabolites and lipids (1). They share properties with NK-cells in that they are capable of killing malignant cells in a non-MHC-restricted manner (2, 3) and have emerged as important players in allogeneic hematopoietic cell transplantation (alloHCT) and immune cell therapy. Prospective studies have shown that the magnitude of TCR-γδ T-cell reconstitution after alloHCT is inversely associated with the incidence of infection (4) and graft-vs.-host disease (GvHD) (5), and is positively associated with leukemia-free and event-free survival (6). The identification of TCR-γδ T-cells as non-alloreactive lymphocytes with potent anti-viral and anti-tumor properties has also seen TCR-γδ T-cells emerge as promising candidates for immunotherapy. The adoptive transfer of *ex vivo* expanded TCR-γδ T-cells has been used successfully to evoke graft- vs.-tumor (GvT) effects against liquid cancers (after alloHCT) such as leukemias and multiple myeloma, and against solid tumors such as renal cell carcinoma, melanoma, and lung cancer (7).

The most widely used method for activating and expanding TCR-γδ T-cells *in vivo* and *ex vivo* is through stimulation with IL-2 and aminobisphosphonates, such as Zoledronate, which preferentially expands the Vγ9Vδ2 subtype (8). However, post-HCT ZOL+IL-2 therapy fails to expand TCR-γδ cells *in vivo* to levels associated with increased survival in ~58% of alloHCT patients (9), while the *ex vivo* expansion of Vγ9Vδ2 with ZOL+IL-2 for adoptive transfer therapy is sometimes unsuccessful due to low numbers of TCR-γδ T-cells in peripheral blood (10). It is important, therefore, to find new ways of mobilizing TCR-γδ T-cells to enrich peripheral blood hematopoietic stem cell grafts prior to transplant, and also to augment TCR-γδ responses to ZOL+IL-2 both *in vivo* and *ex vivo* (9, 11).

One potential target to increase TCR-γδ T-cell mobilization and expansion is the β-adrenergic receptor (β-AR). Indeed, models of systemic β-AR activation in humans such as dynamic exercise, psychosocial stress, and β-agonist (isoproterenol) infusion have been shown to mobilize large numbers of TCR-γδ T-cells to peripheral blood (12–14). While the β-AR could serve as a therapeutic target to increase the proportion of TCR-γδ T-cells in peripheral blood stem cell grafts (e.g., by administering a β-AR agonist to G-CSF mobilized donors), it is not known if systemic β-AR activation will alter the responsiveness of TCR-γδ T-cells to ZOL+IL-2 *ex vivo* or alter the ability of the expanded cells to recognize and kill tumor targets. Moreover, the β-AR subtype (β_1_ vs. β_2_) responsible for their mobilization to the blood and potential augmented expansion and anti-tumor activity is not known.

The aim of this study was to determine if systemic β-AR activation, using acute dynamic exercise as an experimental model, can increase the mobilization, *ex vivo* expansion, and anti-tumor activity of TCR-γδ T-cells isolated from the blood of healthy humans. We also sought to determine the β-AR subtypes involved, by administering a preferential β_1_-AR antagonist (bisoprolol) and a non-preferential β_1_ + β_2_-AR antagonist (nadolol) prior to exercise in a randomized placebo controlled cross-over experiment. We show for the first time that systemic β-AR activation *in vivo* augments the mobilization, *ex vivo* expansion, and anti-tumor activity of TCR-γδ T-cells, and that some of these effects are largely mediated by β_2_-AR signaling and exercise-induced phenotypic shifts that promote a dominant activating signal via NKG2D.

## Methods

### Participants

Fourteen (2 females) healthy cyclists (height: 176.44 ± 2.85 cm, body mass: 77.84 ± 6.91 kg; age: 29.9 ± 6.1 years) volunteered for the first part of this study (Part 1). Participants were excluded if they regularly used any immune modulating medications or tobacco products within the last 6-months, had diagnosed asthma or symptoms of undiagnosed asthma, or had elevated blood pressure, fasting glucose, or fasting cholesterol above normal limits. Participants were required to participate in at least 1–3 h of vigorous exercise per week, corresponding to a score of 5–7 on the Jackson et al. Physical-Activity Rating scale (15). All participants were required to abstain from caffeine consumption and vigorous exercise for 24 h prior to each visit and to arrive at the laboratory following an overnight (8–12 h) fast having consumed only water during this time. A sub-group (*n* = 6; 2 females) of the participants (height: 173.79 ± 9.46 cm, body mass: 72.48 ± 7.84 kg; age: 27.2 ± 3.7 years) also volunteered for the second part of the study (Part 2), which involved taking orally administered β-blockers or placebo prior to exercise. The physiological responses to exercise in the Part 1 group and the Part 2 subgroup are shown in Table 1. All experimental procedures were performed at the Laboratory of Integrated Physiology at the University of Houston and all exercise tests were performed between 07:00 and 10:00. Each participant provided written informed consent and the Committee for the Protection of Human Participants (CPHS) at the University of Houston approved the study.

**Table 1:** Exercise performance and physiological measures of the participants in Part 1 (*n* = 14) and Part 2 (*n* = 6).

	**Part 1 (*n* = 14)**	**Part 2 (*****n*** **=** **6)**			**Trial F (*p*-value)**
		**Placebo**	**Bisoprolol**	**Nadolol**	
Cycling power (watts)	170.71 ± 43.54	150 ± 51.67	150 ± 51.67	150 ± 51.67	ND
Heart rate (bpm)	162.8 ± 11.2	164.5 ± 4.72	127.5 ± 10.84^*^	120.5 ± 8.22^*^	99.954 (*p* < 0.001)
Systolic blood pressure (mmHg)	ND	166.7 ± 10.88	141.33 ± 5.54^*^	137.33 ± 8.52^*^	63.801 (*p* < 0.001)
Diastolic blood pressure (mmHg)	ND	73 ± 7.21	72.33 ± 5.79	73.5 ± 7.48	0.114 (*p =* 0.893)
Lactate (mM)	3.28 ± 1.47	2.27 ± 0.63	2.67 ± 0.88	2.52 ± 0.73	1.534 (*p =* 0.255)
VO_2_ (mL/kg/min)	ND	33.14 ± 10.96	34.8 ± 9.87	35.15 ± 9.15	0.807 (*p =* 0.469)
VE (L/min)	ND	64.72 ± 21.6	72.47 ± 21.2	71.27 ± 22.8	3.567 (*p =* 0.064)
RPE (6–20 Borg Scale)	14.5 ± 1.601	13.83 ± 1.6	15 ± 1.67	15.5 ± 1.87^*^	6.676 (*p =* 0.011)

Measures of heart rate, oxygen uptake (VO_2_), and ventilation (VE) were averaged during the last 5 min of exercise, while rating of perceived exertion (RPE), capillary blood lactate concentration, and blood pressure were recorded immediately prior to exercise cessation. Data are mean ± SD. ND, not determined. Main effects of trial are reported for the Part 2 participants. Statistical differences from placebo are indicated by

* *p <0.05*.

### Experimental Design

The individual blood lactate threshold (BLT) was determined for each participant using a discontinuous graded exercise protocol we have previously described (16). Participants used either their personal road bike mounted to an indoor cycling ergometer (Computrainer, RacerMate Inc., Seattle, W.A.) or a stationary ergometer provided by the laboratory (Velotron, RacerMate Inc.). For the main exercise trial (Part 1), participants completed a 30-min bout of steady state cycling at a power output corresponding to +10 to +15% of their breakpoint BLT (17). Blood was collected before exercise (Pre-Ex), immediately upon exercise cessation (Post-Ex), and 1 h after exercise completion (1 h-Post). Participants who volunteered for the Part 2 component were asked to visit the laboratory on 5 separate occasions at the same time of day with a period of 7-days interspersed between visits. Participants performed a steady state bout of cycling exercise at a power output corresponding to +10% of the individual BLT 3 h after ingesting: (1) 10 mg bisoprolol (preferential β_1_-AR antagonist), (2) 80 mg nadolol (non-preferential β_1_+/β_2_+AR antagonist), or (3) a placebo. Blood samples were collected immediately prior to drug/placebo ingestion (baseline), 3 h later under resting conditions (Pre-Ex), immediately upon cessation of exercise (Post-Ex), and 1 h after exercise completion (1 h Post-Ex). The tablets were administered in a double-blind fashion and the trials were performed using a block randomization design.

### Blood Sampling and Exercise Measures

All intravenous blood samples were collected from an antecubital vein using standard phlebotomy (BD Vacutainer Safety-Lok) and vacutainer tubes spray-coated with sodium-heparin or K_2_EDTA (BD Vacutainer Safety-Lok). The K_2_EDTA tube was used to determine complete blood counts using an automated hematology analyzer (Mindray BC-2800, Nanshan, Shenzen, PR China) and the sodium-heparin tubes for the isolation of peripheral blood mononuclear cells (PBMCs), immunophenotyping, and *ex vivo* expansion of TCR-γδ T-cells. All resting blood samples were collected from the participant following a 10-min period of seated rest. Participants completed a standardized 10-min warm up (< -10% BLT) prior to engaging in the prescribed exercise intensity and were instructed to maintain a consistent pedaling cadence across all exercise trials The Post-Ex blood sample was collected within 3 min of exercise cessation to ensure the mobilized TCR-γδ T-cells were captured (18). Heart rate and respiratory gas exchange were monitored at rest and continuously during exercise by telemetry and indirect calorimetry (Quark CPET, COSMED, Pavona di Albano Laziale, Italy). Ratings of perceived exertion (RPE; Borg Scale) were recorded at rest and every 5-min during exercise. Capillary blood lactate concentration (P-GM7 Micro-Stat Analyzer, Analox instruments Ltd., London, UK) and blood pressure (Part 2 only) were determined at rest and every 10-min during exercise.

### Expansion of TCR-γδ T-Cells

The TCR-γδ T-cells expansion protocol was performed as previously described (8). Briefly, PBMCS were isolated from 10 mL of whole blood collected at Pre-Ex, Post-Ex, and 1 h-Post by density gradient centrifugation (Histopaque-1077, Sigma-Aldrich, St. Louis, MO, USA). PBMCs were counted by flow cytometry and seeded at a concentration of 1 × 10^6^ cells/mL in a 24-well plate with culture media consisting of 100 IU/mL IL-2 and 5 μM of ZOL (Sigma-Aldrich,) in RPMI-1640 (Sigma-Aldrich) with 10% FBS (Sigma-Aldrich) and 1% penicillin streptomycin (Sigma-Aldrich). Media was changed every 3–4 days, with fresh culture media containing 100 IU/mL of IL-2 only (without ZOL). Cells were harvested after 14-days to determine number, phenotype, and function by up to 8-color flow cytometry (MACSQuant 10; Miltenyi Biotec Inc. Bergisch Gladbach, Germany). Expanded TCR-γδ T-cells were enumerated and 2 × 10^5^ cells were labeled with appropriate combinations of the following antibodies, all purchased from eBioscience Inc. (San Diego, CA, USA) unless otherwise stated: CD8-FITC, Vδ2-FITC (BioLegend, San Diego, CA, USA), NKG2C Alexa Flour^®^ 488 (R&D Systems, Minneapolis, MN, USA), CD62L-FITC, CD28-FITC, KLRG1-FITC, CD45RA-FITC, CD3-FITC, TCR-γδ-PE, CD4-PE, CD3-PE, NKG2A-PE (Beckman Coulter, Brea, CA, USA), NKG2D-PE (R&D Systems), CD57-PE, CD27-PE, CD158a-PE (Beckman Coulter), CD158b-PE (Beckman Coulter), CD18e-PE (Beckman Coulter), CD4-PerCP-Cyanine5.5, CD56-PE-Cyanin5.5, CD8-PerCP-Cyanine5.5, PD-1-PerCP-eFluor-710, TCR-γδ-PerCP (BioLegend), CD3-APC, CD56-APC, Vδ1-APC (Miltenyi), NKp30-APC (Biolegend), NKG2A-APC (Beckman Coulter).

### TCR-γδ T-Cell Cytotoxicity Assay With and Without NKG2D Blockade

The HLA-deficient leukemia cell line K562 (ATCC: CCL-243), the HLA-expressing (group 1 HLA-C ^*^0304,^*^0702) multiple myeloma cell line U266 (ATCC:TIB-196), and the 221.AEH (a HLA-E+ transfectant derived from the 721.221 lymphoma cell line) (19) were maintained as previously described and used as target cells for the cytotoxicity assay (16). On the day of the TCR-γδ T-cells cytotoxicity assay, 3 × 10^6^ target cells were removed and labeled with anti-CD71-FITC and co-cultured with the expanded TCR-γδ T-cells at 0:1 (determine spontaneous death), 1:1, 5:1, 10:1, and 20:1 TCR-γδ T-cell: target cell ratios. All assays were conducted in the presence of RPMI-1640 with 10% FBS and 1% penicillin streptomycin and incubated at 37°C for 4 h in a humidified CO_2_ incubator. Flow cytometry was used to determine TCR-γδ T-cell cytotoxic activity following similar methods we have described previously for NK-cells (20). TCR-γδ T-cell cytotoxic activity was calculated as specific lysis (% total lysis – % spontaneous death) and lytic index (number of dead target cells per 100,000 TCR-γδ T-cells). To block activation of TCR-γδ T-cells through the NKG2D receptor, TCR-γδ T-cells were incubated with either media alone, an REA isotype control, or an anti-NKG2D monoclonal antibody (REA, clone REA797) for 30 min prior to performing the TCR-γδ T-cell cytotoxicity assay. Finally, NKG2D ligands were measured on K562 and U266 cells lines using monoclonal antibodies against the following targets: MICA/MICB (Miltenyi), ULBP-2/5/6 (R&D Systems), ULBP-1 (R&D Systems), and ULBP-3 (R&D Systems).

### Statistical Analysis

All statistical analyses were completed using SPSS (v24.0, IBM, Chicago, IL). Maximum Likelihood, linear mixed models (LMM) were used to analyze the numeric changes in expansions, cytotoxicity, and phenotypes. The LMM allowed for modeling the dependencies within persons from having multiple measures per person. For Part 1, the model included main effects for exercise time (Pre-Ex, Post-Ex, and 1 h-Post). The effect of NKG2D blockade on TCR-γδ T-cell killing against U266 and K562 was determined by two-way repeated measures ANOVA that included main effects of exercise time (pre-ex and post-ex) and condition (media alone, isotype control, and anti-NKG2D). The model for Part 2 included main effects for exercise time and trial (placebo, bisoprolol, and nadolol), as well as an interaction effect for exercise time and trial. Planned contrasts were used *a priori* to determine overall time effects within each trial (placebo, bisoprolol, or nadolol), the location of the significant time effects (Pre-Ex, Post-Ex, and 1 h-Post), and significant differences across the three trials at each specified time point. All data are represented as the mean ± SD unless otherwise stated and significance was set at *p* < 0.05.

## Results

### Acute Exercise Mobilizes TCR-γδ T-Cells in a β_2_-AR Dependent Manner

**Figure 1:**
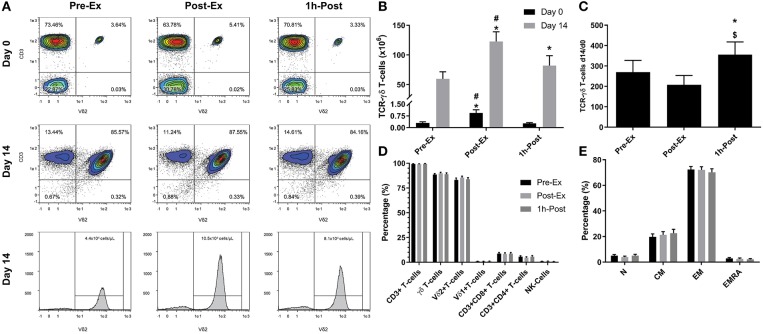
Exercise augments the *ex vivo* expansion of Vγ9δ2 T-cells: **(A)** representative flow cytometry contour plots and histograms showing the effect of exercise on the proportions of Vγ9δ2 T-cells among total PBMCs at Day 0 and after 14-days expansion with ZOL+IL-2. Histogram events are adjusted to reflect the total number of cells isolated per μI of cell culture supernatant. **(B)** The total number of TCR-γδ cells in 1 × 10^6^ PBMCs isolated before (Pre-Ex), immediately after (Post-Ex), and 1 h after exercise (1 h-Post) at Day 0, and the total number of TCR-γδ T-cells generated in the expanded cell products after 14-days stimulation with ZOL+IL-2. Significant differences were only determined within expansion period (Day 0 or 14) and not between. **(C)** The number of TCR-γδ cells generated at Day 14 divided by the number of TCR-γδ cells in the PBMC fractions at Day 0. **(D)** The cellular composition of the expanded TCR-γδ T-cell products at Day 14. **(E)** The proportions of naïve (N), central memory (CM), effector memory (EM), and CD45RA+ effector memory (EMRA) cells among total TCR-γδ T-cells. Values are mean ± SE (*n* = 14). Differences from Pre-Ex, Post-Ex, and 1 h-Post are indicated by *, $, and #, respectively (*p* < 0.05).

**Figure 2:**
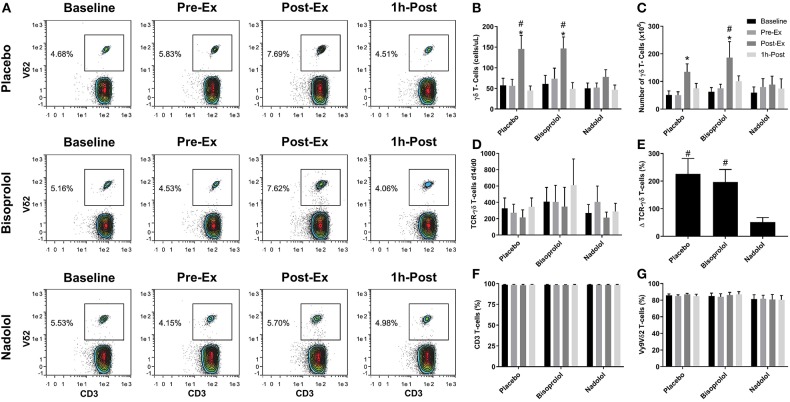
β_1_ + β_2_-AR but not β_1_-AR blockade alone inhibits the mobilization and augmented *ex vivo* expansion of Vγ9δ2 T-cells in response to exercise. **(A)** Representative flow cytometry contour plots showing the effects of a β_1_ + β_2_-AR antagonist (nadolol) and a β_1_-AR antagonist (bisoprolol) on the mobilization of Vγ9δ2 T-cells with exercise. Values shown are the percentage of all CD3+ T-cells. **(B)** The total numbers of TCR-γδ T-cells in blood before and after exercise. After 14 days' expansion with ZOL+IL-2, **(C)** the total number, **(D)** the number of TCR-γδ cells generated at Day 14 divided by the number of TCR -γδ cells in the PBMC fractions at Day 0 and **(E)**, percentage change in number of TCR-γδ T-cells expanded Post-Ex relative to baseline. The percentage of **(F)** CD3+ and **(G)** Vγ9Vδ2+ T-cells among the expanded cell products are shown. Values are mean ± SE (*n* = 6). Significant difference from Baseline/Pre-Ex/1 h-Post and the nadolol trial indicated by * and #, respectively (*p* < 0.05).

First, we examined the effect of exercise on the mobilization of TCR-γδ T-cells to the peripheral blood compartment (Table 2 and Figure 1A). The absolute number (*p* < 0.001; Table 2) and percentage (*p* < 0.001; Figure 1A) of TCR-γδ T-cells was elevated in blood Post-Ex compared to Pre-Ex and 1 h-Post (*p* < 0.01). When examining the effects of bisoprolol and nadolol on the mobilization of TCR-γδ T-cells after exercise relative to placebo, a main effect of exercise time (*p* < 0.001) and an interaction between exercise time and trial was found for both the absolute number and percentage of TCR-γδ T-cells among CD3+ T-cells (*p* < 0.01; Table 2 and Figures 2A,B). Planned contrasts showed that nadolol but not bisoprolol blunted the effects of exercise on TCR-γδ T-cell mobilization to the blood, indicating the mobilization of TCR-γδ T-cells is dependent on β_2_-AR signaling. Neither nadolol nor bisoprolol inhibited the exercise-induced mobilization of total lymphocytes, total CD3+ T-cells, or CD4+ T-cells. Significant time × trial interactions were found for the mobilization of CD8+ T-cells, CD3+/CD4–/CD8– T-cells, and NK-cells with the effect sizes for time were lowest in the nadolol trial (Table 2).

**Table 2:** The total number (cells/μL) of lymphocytes, CD3+ T-cells, CD4+ T-cells, CD8+ T-cells, CD4–/CD8– T-cells, Vγ9Vδ2 T-cells, and NK-cells (CD3–CD56+) before, immediately after, and 1 h after exercise for the participants in Part 1 (*n* = 14) and Part 2 (*n* = 6).

		**Baseline**	**Pre-Ex**	**Post-Ex**	**1 h-Post**	**Time effect F (*p*-value)**	**Main effects**		
							**Time**	**Trial**	**Interaction**
**LYMPHOCYTES**									
**Part 1**		ND	1617.9 ± 245.4	3135.7 ± 664.9^#~^	1641.7 ± 365.5	F = 80.37 (<0.001)			
**Part 2**	Placebo	1816.7 ± 625.03	1700 ± 327.1	3025 ± 479.3^*^^#~^	1810 ± 386.3	F = 16.71 (<0.001)	F = 43.95	F = 0.45	F = 0.76
	Bisoprolol	1791.7 ± 442.1	2016.7 ± 388.2	3183.3 ± 677.3^*^^#~^	1760 ± 451.9	F = 18.99 (<0.001)	*p* < 0.001	*p =* 0.643	*p* = 0.602
	Nadolol	1675 ± 448.1	1808.3 ± 327.8	2716.7 ± 805.4^*^^#~^	1790 ± 449.2	F = 9.77 (<0.001)			
**Trial effect:**		F = 0.16	F = 0.73	F = 1.6	F = 0.03				
		*p* = 0.851	*p* = 0.485	*p* = 0.213	*p* = 0.971				
**CD3+** **T-CELLS**									
**Part 1**		ND	1066.2 ± 224.2	1785.5 ± 448.6^#~^	1199 ± 263.3^*^	F = 39.62 (<0.001)			
**Part 2**	Placebo	1306.9 ± 483.4	1188.5 ± 225.6	1789.5 ± 356.8^*^^#~^	1322.3 ± 274.3^*^	F = 11.86 (<0.001)	F = 28.84	F = 0.21	F = 1.21
	Bisoprolol	1313.1 ± 444.9	1461 ± 361.2	1908.5 ± 476.2^*^^#~^	1349.8 ± 412.8^*^	F = 13.18 (<0.001)	*p* < 0.001	*p* = 0.816	*p* = 0.316
	Nadolol	1216.8 ± 421.9	1332.3 ± 326.2	1666 ± 442.5^*^^#~^	1355.9 ± 411.2^*^	F = 6.23 (0.001)			
**Trial effect:**		F = 0.14	F = 0.88	F = 0.7	F = 0.02				
		*p* = 0.872	*p* = 0.426	*p* = 0.507	*p* = 0.976				
**CD4+** **T-CELLS**									
**Part 1**		ND	646.9 ± 169	1026.1 ± 325^#~^	760.2 ± 222.5	F = 25.94 (<0.001)			
**Part 2**	Placebo	826.5 ± 371.5	722 ± 188.5	989.7 ± 265.2^#^	847.7 ± 240.7	F = 5.48 (0.002)	F = 11.8	F = 0.04	F = 1.02
	Bisoprolol	832.8 ± 374.2	889.3 ± 237	1001.4 ± 203^~^	850.9 ± 285.6	F = 3.17 (0.032)	*p* < 0.001	*p* = 0.959	*p* = 0.424
	Nadolol	767.9 ± 330.1	823.9 ± 247.4	1014.4 ± 359.5^*^^#^	841.7 ± 317.7	F = 5.17 (0.003)			
**Trial effect:**		F = 0.11	F = 0.62	F = 0.01	F = 0.01				
		*p* = 0.895	*p* = 0.549	*p* = 0.987	*p* = 0.987				
**CD8+** **T-CELLS**									
**Part 1**		ND	338.1 ± 117.7	582.9 ± 186^#~^	358.8 ± 129	F = 36.8 (<0.001)			
**Part 2**	Placebo	385.6 ± 178.7	367 ± 148.6	588.2 ± 186.4^*^^#~^	373.7 ± 165	F = 11.42 (<0.001)	F = 31.64	F = 0.24	F = 2.35
	Bisoprolol	385.7 ± 171.8	460.3 ± 257.3	694.8 ± 379.6^*^^#~^	405.7 ± 228.3	F = 21.19 (<0.001)	*p* < 0.001	*p* = 0.783	*p* = 0.045
	Nadolol	359.2 ± 146.3	402.2 ± 163.4	500.8 ± 153.9^*^^#~^	410.4 ± 181.6	F = 3.720 (0.017)			
**Trial effect:**		F = 0.04	F = 0.387	F = 1.644	F = 0.03				
		*p* = 0.960	*p* = 0.683	*p* = 0.215	*p* = 0.974				
**CD4–/CD8– T-CELLS**									
**Part 1**		ND	77.4 ± 37.3	166.9 ± 85.6^#~^	75.2 ± 35.6	F = 26.95 (<0.001)			
**Part 2**	Placebo	91.3 ± 47.2	95.8 ± 43.5	205.1 ± 100.8^*^^#~^	95.6 ± 36.7	F = 21.85 (<0.001)	F = 43.33	F = 0.17	F = 2.43
	Bisoprolol	90.5 ± 52.9	106.3 ± 60.7	207.2 ± 69.6^*^^#~^	88.5 ± 41.7	F = 21.93 (<0.001)	*p* < 0.001	*p* = 0.845	*p* = 0.038
	Nadolol	87 ± 55.4	101.8 ± 44.1	145 ± 59.4^*^	97.8 ± 53.8	F = 4.42 (0.008)			
**Trial effect:**		F = 0.01	F = 0.06	F = 2.71	F = 0.08				
		*p* = 0.989	*p* = 0.942	*p* = 0.083	*p* = 0.920				
**Vγ9Vδ2 T-CELLS**									
**Part 1**		ND	42.8 ± 32.9	115.6 ± 67.6^#~^	37.9 ± 26.1	F = 31.32 (<0.001)			
**Part 2**	Placebo	57.5 ± 42	56.5 ± 38.4	145.6 ± 80.5^*^^#~^^$^	44.9 ± 24.2	F = 22.36 (<0.001)	F = 36.33	F = 0.43	F = 3.99
	Bisoprolol	58 ± 49.6	70.3 ± 61.3	147 ± 67.3^*^^#~^^$^	47.9 ± 34	F = 20.37 (<0.001)	*p* < 0.001	*p* = 0.657	*p* = 0.002
	Nadolol	52.3 ± 40.4	56.7 ± 33.2	79.4 ± 47	53.3 ± 32.2	F = 1.58 (0.206)			
**Trial effect:**		F = 0.03	F = 0.19	F = 4.53	F = 0.07				
		*p* = 0.97	*p* = 0.829	*p* = 0.02	*p* = 0.935				
**CD3–CD56+**									
**Part 1**		ND	185.5 ± 62.2	746.3 ± 244.9^#~^	121 ± 44.3	F = 71.41 (<0.001)			
**Part 2**	Placebo	151 ± 66.6	161.6 ± 64.9	657.2 ± 312.1^*^^#~^^$^	94.2 ± 24.5	F = 17.23 (<0.001)	F = 37.66	F = 2.21	F = 2.41
	Bisoprolol	152 ± 53.6	183.4 ± 75.1	732.5 ± 433.2^*^^#~^^$^	94.1 ± 28.2	F = 22.01 (<0.001)	*p* < 0.001	*p* = 0.141	*p* = 0.040
	Nadolol	145.2 ± 51.4	130.3 ± 49.7	343.7 ± 252.9^*^^#~^	74.7 ± 19.8^*^	F = 3.24 (0.03)			
**Trial effect:**		F = 0.003	F = 0.16	F = 9.4	F = 0.02				
		*p* = 0.997	*p* = 0.855	*p* < 0.001	*p* = 0.982				

Baseline blood samples were collected from the part 2 participants prior to drug/placebo administration then again 3 h later immediately prior to exercise onset (Pre-Ex). For the part 2 participants, main effects of time, trial, and time x trial interaction effects are shown with the planned contrasts for time and trial. Data are mean ± SD. ND, not determined. Differences from baseline, Pre-Ex, and 1 h post are indicated by

* , #, ~, respectively (p <0.05). Differences from the nadolol trial are indicated by

$ *(p <0.05)*.

### Acute Exercise Augments the *ex vivo* Expansion of TCR-γδ T-Cells and Is Dependent on β_2_-AR Signaling

We examined the effect of exercise on the expansion of TCR-γδ T-cells (Figure 1). As expected, a greater number of TCR-γδ T-cells were present in PBMCs isolated Post-EX compared to both Pre-Ex and 1 h Post-Ex PBMCs (*p* < 0.001; Figure 1B). Following 14-day's expansion with ZOL+IL-2, significantly more TCR-γδ T-cells were generated from the PBMCs collected Post-Ex compared to PBMCs collected at baseline/Pre-Ex and 1 h-Post (*p* < 0.001; Figure 1B). Also, PBMCs collected at 1 h-Post generated significantly more TCR-γδ T-cells than PBMCs collected at baseline/Pre-Ex (*p* < 0.05; Figure 1B). The number of TCR-γδ T-cells generated after 14-days from each TCR-γδ T-cell stimulated on Day-0 was highest from PBMCs collected at 1 h-Post compared to Pre-Ex and Post-Ex (*p* < 0.01; Figure 1C). The composition of the expanded TCR-γδ T-cells was primarily Vγ9Vδ2 T-cells (>93%) and exercise had no effect on the percentage and differentiation of Vγ9Vδ2 T-cells among the expanded cell products (*p* > 0.05; Figures 1D,E). When examining the effects of bisoprolol and nadolol relative to placebo on the expansion of TCR-γδ T-cells after exercise (Figure 2), a main effect of exercise time (*p* < 0.001) and an interaction between exercise time and trial was found (*p* = 0.018; Figures 2C,E). Planned contrasts showed that nadolol blunted the exercise-induced increase in TCR-γδ T-cell expansion but bisoprolol did not (Figure 2E). Neither nadolol nor bisoprolol affected the percentage of CD3+ T-cells, total TCR-γδ T-cells or Vγ9Vδ2 T-cells among the expanded cell products (Figures 2F,G).

### Acute Exercise Drives Expanded TCR-γδ T-Cells Toward an Activated Phenotype With Anti-Tumor and Tissue Migration Potential

**Figure 3:**
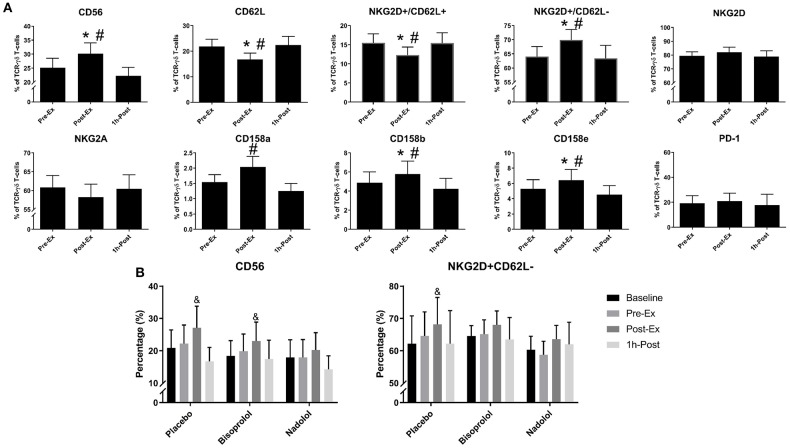
Exercise alters the surface phenotypes of *ex vivo* expanded TCR-γδ T-cells. **(A)** The percentage of CD56+, CD62L+, NKG2D+/CD62L+, NKG2D+/CD62L–, NKG2D+, NKG2A+, CD158a+, CD158b+, CD158e+, and PD-1+ cells among total TCR-γδ T-cells after 14-days expansion (*n* = 14). **(B)** The effects of administering a placebo, bisoprolol, or nadolol prior to exercise on the proportions of CD56+ and NKG2D+/CD62L– cells among total TCR-γδ T-cells after 14-days expansion (*n* = 6). Values are mean ± SE. Significant differences from baseline, Pre-Ex, and 1 h-Post are indicated by &,*, and #, respectively (*p* < 0.05).

We performed a detailed phenotypic analysis of the expanded TCR-γδ T-cell products, focusing on the expression of surface markers associated with activation, inhibition, homing, differentiation, and exhaustion (Figure 3). We found that Post-Ex expanded TCR-γδ T-cells had higher proportions of CD56+ (*p* < 0.01), NKG2D+/CD62L– (*p* < 0.01), CD158b+ (*p* < 0.05), and CD158e+ (*p* < 0.05) and decreased proportions of NKG2A+ (*p* < 0.05), CD62L+ (*p* < 0.001), NKG2D+/CD62L– (*p* < 0.01) compared to Pre-Ex and 1 h-Post expanded TCR-γδ T-cells (Figure 3A). There was no effect of exercise (*p* > 0.05) on the proportions of TCR-γδ T-cells expressing PD-1, indicating that the augmenting effects of exercise did not drive the TCR-γδ T-cell product to exhaustion after 14-days expansion (Figure 3A). Administering nadolol but not bisoprolol prior to exercise inhibited the phenotypic shifts seen in the expanded TCR-γδ T-cell products, particularly for CD56+ (Figure 3B). Conversely, the proportion of Post-Ex NKG2D+/CD62L– expanded TCR-γδ T-cells was inhibited by administration of bisoprolol or nadolol prior to exercise (Figure 3B). These results indicate that exercise drives phenotypic changes within the expanded TCR-γδ T-cell products that are associated with increased cytotoxicity and tissue migration potential in a manner that is also dependent on β_2_-AR signaling.

### Acute Exercise Augments the *in vitro* Cytotoxicity of the Expanded TCR-γδ T-Cell Products in a Manner That Is Dependent on Both β_1_-AR and β_2_-AR Signaling

**Figure 4:**
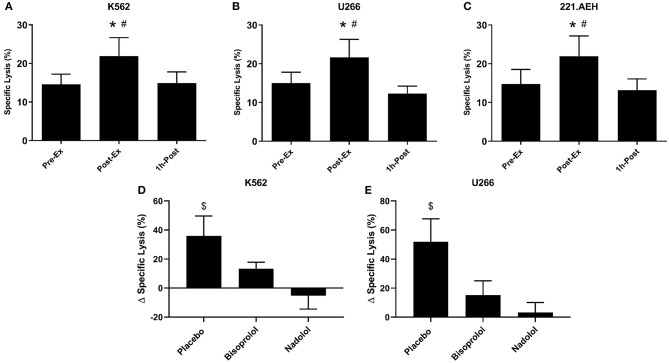
Exercise augments the anti-tumor activity of *ex vivo* expanded TCR-γδ T-cells in a manner that is dependent on both β_1_ and β_2_-AR signaling. The percentage of K562 **(A)**, U266 **(B)**, and 221.AEH **(C)** target cells killed by the expanded TCR-γδ T-cells at an E:T ratio of 10:1 following 14-days expansion with ZOL+IL-2 (*n* = 14). The effects of placebo, bisoprolol, and nadolol on the exercise-induced changes in specific lysis against K562 **(D)** and U266 **(E)** target cells following *ex vivo* expansion (*n* = 6). Values are mean ± SE. Differences from Pre-Ex are indicated by * and differences from 1 h-Post are indicated by #, *p* < 0.05. Differences from the nadolol trial are indicated by $, *p* < 0.05.

We examined the function of expanded TCR-γδ T-cells by determining their *in vitro* cytotoxicity against three different tumor cells lines, K562, U266, and 221.AEH (Figure 4). The TCR-γδ T-cells expanded Post-Ex had increased cytotoxic activity against all tumor targets compared to TCR-γδ T-cells expanded at both Pre-Ex and 1 h-Post (*p* < 0.01; Figures 4A–C). This finding held true when data were expressed as specific lysis, lytic index, and total killing capacity (data not shown). The effects of nadolol and bisoprolol on the cytotoxicity of TCR-γδ T-cells expanded after exercise were assessed for the K562 and U266 cell lines only. Due to the small sample size and large intrasubject variability, these data are expressed as a percentage change in killing capacity of the Post-Ex expanded TCR-γδ T-cells compared to those expanded Pre-Ex (Figures 4D,E). Compared to placebo, nadolol abrogated the exercise-induced increase in the cytotoxic activity of the expanded TCR-γδ T-cell products against both the K562 and the U266 cell line. Bisoprolol was found to inhibit the exercise effects on the cytotoxic activity of the expanded TCR-γδ T-cells, but not to the same extent as nadolol. These findings indicate that TCR-γδ T-cells expanded immediately after exercise have increased anti-tumor activity compared to TCR-γδ T-cells expanded at rest and during the early (1 h-Post) stage of exercise recovery, and that the exercise effects are dependent on both β_1_-AR and β_2_-AR signaling.

### Exercise Increases TCR-γδ T-Cell Cytotoxic Activity Against U266 Target Cells Through NKG2D Dependent Signaling

**Figure 5:**
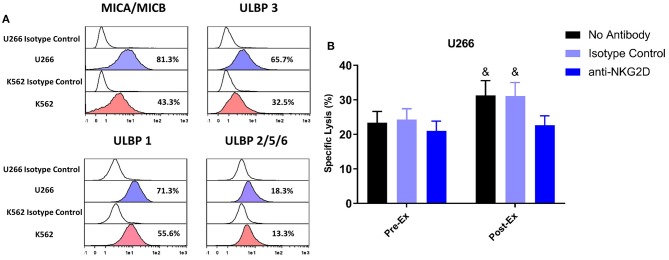
Enhanced cytotoxic effect of exercise expanded TCR-γδ T-cells against U266 cells is dependent on NKG2D signaling. **(A)** The expression of NKG2D ligands, MICA/MICB, ULBP 3, ULBP 1, and ULBP 2/5/6 on U266 and K562 target cell lines. **(B)** The percentage of U266 cells killed by Pre-Ex and Post-Ex expanded TCR-γδ T-cells under 3 different conditions: no antibody (media), isotype control (REA), and anti-NKG2D. Values are mean ± SE. Differences from Post-Ex anit-NKG2D condition are indicated by &, *p* < 0.05.

A greater proportion of TCR-γδ T-cells expanded Post-Ex displayed an NKG2D+/CD62L– phenotype compared to those expanded at Pre-Ex. We, therefore, examined the effects of blocking the NKG2D receptor on TCR-γδ T-cells prior to performing the *in vitro* cytotoxicity assay against U266 and K562 tumor cells. NKG2D blockade on Pre-Ex expanded TCR-γδ T-cells did not alter cytotoxic activity against U266 cells (p > 0.05) but the enhanced cytotoxic effect of TCR-γδ T-cells expanded Post-Ex was abrogated by NKG2D blockade (p <0.05; Figure 5B). Blocking NKG2D had no impact on the exercise-induced increase in TCR-γδ T-cell cytotoxicity against K562 tumor cells (data not shown). Tellingly, the U266 cell line had a greater surface expression of the NKG2D ligands MICA/MICB, ULBP-1, and ULBP-3 compared to K562 cells (Figure 5A).

## Discussion

TCR-γδ T-cells have emerged as an important subset of effector lymphocytes in allo-HCT and adoptive transfer immunotherapy due to their ability to attack a wide range of solid and hematological tumors without causing GvHD. However, the low frequency of TCR-γδ T-cells in the peripheral blood of healthy donors limits their therapeutic potential. We have demonstrated that systemic β-AR activation, using acute dynamic exercise as an experimental model, mobilizes TCR-γδ T-cells to the peripheral blood and, for the first time here, augments their *ex vivo* expansion and anti-tumor activity. These adjuvant effects of exercise were associated with certain phenotypic shifts and found to be largely dependent on β_2_-AR signaling, as they were abrogated by administering a β_1_ + β_2_-AR antagonist (nadolol) but not a β_1_-antagonist (bisoprolol) prior to exercise. These findings highlight the β_2_-AR as a potential therapeutic target to mobilize TCR-γδ T-cells in healthy alloHCT donors and to also increase the potency of TCR-γδ T-cell therapeutics.

Prior studies have demonstrated that systemic β-AR activation using either exercise or isoproterenol infusion, increases the number of circulating TCR-γδ T-cells 2–4-fold (12, 14). We have shown previously that exercise has potent adjuvant effects on the mobilization and *ex vivo* manufacture of viral specific T-cells and were interested to evaluate whether these findings extend to TCR-γδ T-cells also (21–23). This is particularly pertinent as TCR-γδ T-cell reconstitution after alloHCT has been associated with improved leukemia free survival and protection against GvHD (4–6). The *ex vivo* manufacture and adoptive transfer of TCR-γδ T-cells are being applied to elicit GvT effects in alloHCT recipients and in the treatment of solid tumors such as renal cell carcinoma, melanoma, lung cancer, and neuroblastoma (7). We determined that just 30-min of intensity-controlled exercise increased the numbers of TCR-γδ T-cells in peripheral blood almost 3-fold (~Δ 73 cells/μL). This allowed us to manufacture 2-times more TCR-γδ T-cells from blood samples collected during exercise compared to rest. Moreover, the TCR-γδ T-cells expanded after exercise had a greater surface expression of several activating receptors, a lowered expression of inhibitory receptors, and were found to have superior cytolytic activity *in vitro* against various tumor targets than TCR-γδ T-cells expanded at rest. The limited therapeutic application of TCR-γδ T-cells to date has been attributed to low circulating numbers in healthy transplant donors (1–5% of blood lymphocytes) (8). This results in low numbers of TCR-γδ T-cells in peripheral blood stem cell grafts of G-CSF mobilized donors, which could be partially responsible for poor TCR-γδ T-cell reconstitution in alloHCT patients posttransplant (4, 24). Using systemic β-AR activation to enrich G-CSF mobilized peripheral blood stem cell grafts with TCR-γδ T-cells could also improve *ex vivo* graft engineering methods such as TCR-αβ+CD19 depletion (25), which are designed to protect against GvHD and EBV-induced lymphoproliferative disease but retain TCR-γδ T-cells and NK-cells in the graft to maintain anti-tumor activity. This could help overcome some reported limitations with this method such as graft failure and poor immune reconstitution, which leads to an increased incidence of relapse and infection (26). Moreover, although ZOL+IL-2 has been used to expand TCR-γδ T-cells (Vδ2 subset) *in vitro* and *in vivo*, TCR-γδ T-cells respond poorly to ZOL+IL-2 in some patients and donors (8, 10, 27–30). The current findings indicate that systemic β-AR activation is a simple, economical, and effective adjuvant for the potential enrichment of peripheral blood stem cell grafts with TCR-γδ T-cells, and also for increasing their responsiveness to exogenous cytokines and phosphoantigens such as ZOL+IL-2. This could be effective, not only for the *ex vivo* manufacture of TCR-γδ T-cell products shown here, but also for expanding TCR-γδ T-cells *in vivo* following alloHCT using subcutaneous ZOL+IL-2 injections.

Our finding that the adjuvant effects of exercise are largely dependent on β_2_-AR signaling has important practical applications as it might allow us to further optimize potential pharmaceutical interventions for mobilizing and expanding TCR-γδ T-cells in healthy donors. Administering a preferential β_1_-AR antagonist (e.g., bisoprolol) prior to exercise or isoproterenol infusion is likely to provide the required β_2_-AR stimulation to mobilize and activate TCR-γδ T-cells without causing sustained elevations in heart rate and systolic blood pressure, which is predominantly a β_1_-AR mediated effect (31). Indeed, we found that both bisoprolol and nadolol blunted the exercise-induced increases in heart rate and blood pressure but the adjuvant effects of exercise were still apparent in the bisoprolol trial. This finding likely excludes increases in hemodynamic shear stress as a potential mechanism for the adjuvant effects of exercise on TCR-γδ T-cell mobilization and *ex vivo* expansion. Bisoprolol did, however, blunt the effects of exercise on the cytotoxic capabilities of the expanded TCR-γδ T-cell product suggesting a role for both β_1_-ARs and β_2_-ARs in improving the function of *ex vivo* expanded TCR-γδ T-cells. An important observation was the augmented *ex vivo* expansion of the TCR-γδ T-cells expanded from blood collected 1 h after exercise cessation compared to rest. As the numbers of TCR-γδ T-cells in blood at this time were lower than at rest, this indicates that the adjuvant effects of exercise are not merely due to increased numbers of TCR-γδ T-cells within a fixed volume of blood.

Previous studies have shown that ZOL+IL-2 expands a TCR-γδ T-cell product with a high surface expression of the activating receptors NKG2D, TRAIL, DNAM-1, an increased expression of perforin and granzyme-B, and transforms the cells to an effector/central memory phenotype (8, 10, 32). Regardless of whether blood samples were collected at rest, during, or in the recovery phase of exercise, the 14-day *ex vivo* expansion with ZOL+IL-2 generated a cell product that was >98% pure for CD3+ T-cells. This expanded cell product consisted primarily of CD3+/CD4–/CD8– Vγ9Vδ2 T-cells with few CD8+ and CD4+ T-cells and almost no NK-cells (<1%). Similarly, administering nadolol or bisoprolol prior to exercise had no impact on the purity of the expanded TCR-γδ T-cell product. However, we did find some phenotypic differences in the TCR-γδ T-cell products expanded from exercised compared to resting blood. Notably, greater proportions of NKG2D+/CD62L−, CD56+, CD158a+, CD158b+, and CD158e+ and decreased proportions of NKG2A+ and CD62L+ cells were found among the Vγ9Vδ2+ T-cells expanded after exercise. As TCR-γδ T-cells recognize stress-associated antigens such as MICA/B and nectin-like-5 via NKG2D and DNAM-1, respectively (33), it is possible that these phenotypic changes on the expanded cell product with exercise are responsible for the augmented cytotoxic effects seen against the K562, U266, and 221.AEH cell lines *in vitro*. Indeed, although expression of the activating receptor NKG2D was not upregulated by exercise on the expanded TCR-γδ T-cells, blocking NKG2D *in vitro* abrogated the enhanced cytotoxic effects of the TCR-γδ T-cells expanded after exercise against U266 target cells. This indicates that exercise evokes phenotypic shifts (mainly a downregulation of inhibitory receptors) that allow activation signals via NKG2D to be more dominant. However, blocking NKG2D did not eliminate the enhancing effects of exercise on the cytotoxic effects of TCR-γδ T-cells against the K562 target cell line. This could be due to our U266 target cells expressing higher levels of the NKG2D ligands MICA/MICB ULBP-1 and ULBP-3 than the K562 cells, corroborating what has been published previously (34, 35). Moreover, nadolol prevented some of the phenotypic shifts seen with exercise (e.g., CD56+ and NKG2D+/CD62L–) concomitantly with an abrogation of the exercise-induced improvements in TCR-γδ T-cell cytotoxicity. It remains possible that the enhanced cytotoxic effects of TCR-γδ T-cells against K562 target cells with exercise are due to phenotypic shifts we did not capture in our study. Also, because we only tested the effects of β-AR blockade on a subgroup of participants, this might explain why nadolol did not abrogate all of the phenotypic changes observed during the main exercise trial to a level that reached statistical significance.

Exercise is known to preferentially mobilize TCR-γδ T-cells with a differentiated phenotype, increasing proportions of effector memory (EM), CD45RA+ EM (EMRA), and central memory (CM) cells among total TCR-γδ T-cells in blood (16, 36, 37). However, expanding TCR-γδ T-cells *ex vivo* overrode these phenotypic shifts of exercise, as similar proportions of naïve (~5%), EM (~71%), CM (~21%), and EMRA (~3%) cells were found among the expanded Vγ9Vδ2+ T-cells regardless of whether blood samples were collected before, during, or in the recovery phase of exercise. Previous studies have shown that IPP and Zoledronate stimulation causes a shift toward an EM phenotype in expanded Vγ9Vδ2+ T-cells and these cells have enhanced cytotoxic activity and increased IFN-γ release in response to activation (8, 10, 38, 39).

A limitation of the present study is that we only attempted to expand Vγ9Vδ2+ and not Vδ1+ T-cells with exercise. Although Vγ9Vδ2+ T-cells are the most abundant subset of TCR-γδ T-cells in blood, the Vδ1+ subset is known to play a prominent role in anti-tumor immunity and in the resolution of viral infections such as cytomegalovirus (40). We did not expect to find increased numbers of Vδ1+ T-cells in our expanded products as this subset of TCR-γδ T-cells do not respond to aminobisphosphonates. There are methods for the *ex vivo* expansion and adoptive transfer of Vδ1+ cells has been used in the treatment of multiple myeloma (41), colon cancer (42), B-cell chronic lymphocytic leukemia (43), and glioblastoma (44), but it remains to be seen if systemic β-AR activation can augment the *ex vivo* manufacture of this cell type also. It is also possible that the adjuvant effects of exercise on the *ex vivo* expansion of TCR-γδ T-cells are monocyte-dependent. Previous studies have shown that removing monocytes from culture results in no expansion of TCR-γδ T-cells when stimulated with ZOL+IL-2 (45). When aminobiphosphates such as ZOL are taken up by monocytes, they block farnesyl pyrophosphate synthase (FPPS) resulting in an intracellular accumulation of isopentenyl phosphate (IPP) via the mevalonate pathway. Subsequent cell-to-cell contact then triggers the activation and expansion of Vγ9Vδ2 T-cells (46). Exercise is known to cause proportional and phenotypic changes within the blood monocyte population (18, 47–50) and it is possible that these are driving the adjuvant effects of exercise on the *ex vivo* expansion and increased cytotoxicity of TCR-γδ T-cells. Specifically, exercise increases the proportions and absolute number of non-classical monocytes, which release higher levels of pro inflammatory cytokines (e.g., TNF-α and IL-18) that are essential for the activation and expansion of Vγ9Vδ2 T-cells (51). Moreover, the activation of Vγ9Vδ2 T-cells by ZOL and IPP requires expression of butyrophilin BTN3A molecules on monocytes (52), which could also be affected by exercise. Future studies should determine the role monocytes play in activating and expanding TCR-γδ T-cells after exercise. It will also be important to determine if the TCR-γδ T-cells expanded with systemic β-AR activation persist following adoptive transfer and exert improved anti-tumor effects *in vivo*.

In summary, this is the first study to show that systemic β-AR activation augments the *ex vivo* expansion and anti-tumor cytotoxicity of Vγ9Vδ2 T-cells in healthy humans. We have also demonstrated that these effects are largely mediated by catecholamine signaling through the β_2_-AR subtype and exercise-induced phenotypic shifts among the expanded Vγ9Vδ2 T-cells that promote a dominant activating signal through the NKG2D receptor. Future studies should determine if targeted β_1_-AR and/or β_2_-AR activation in allogeneic donors can generate superior G-CSF mobilized peripheral stem cell grafts and increase the potency of TCR-γδ T-cell therapeutics to elicit more positive outcomes in alloHCT patients and patients with solid tumors.

## Data Availability Statement

The datasets generated for this study are available on request to the corresponding author.

## Ethics Statement

The studies involving human participants were reviewed and approved by Committee for the Protection of Human Participants (CPHS) at the University of Houston. The patients/participants provided their written informed consent to participate in this study.

## Author Contributions

RS, CB, RB, and FB developed the theoretical framework, working hypotheses, and designed the study. FB, AB, and NA performed the laboratory experiments and analyzed results. FB, RS, AB, CB, EK, RB, CP, and DO'C interpreted data. FB and RS wrote the manuscript with contributions from AB, CB, EK, DO'C, CP, and RB. The statistical analyses were performed by FB and DO'C. The overall study was supervised by RS.

### Conflict of Interest

The authors declare that the research was conducted in the absence of any commercial or financial relationships that could be construed as a potential conflict of interest.
